# An actinobacteria lytic polysaccharide monooxygenase acts on both cellulose and xylan to boost biomass saccharification

**DOI:** 10.1186/s13068-019-1449-0

**Published:** 2019-05-10

**Authors:** Thamy Lívia Ribeiro Corrêa, Atílio Tomazini Júnior, Lúcia Daniela Wolf, Marcos Silveira Buckeridge, Leandro Vieira dos Santos, Mario Tyago Murakami

**Affiliations:** 1 Brazilian Bioethanol Science and Technology Laboratory (CTBE), Brazilian Center for Research in Energy and Materials (CNPEM), Campinas, SP Brazil; 2 Institute of Biosciences, University of São Paulo (USP), São Paulo, Brazil

**Keywords:** LPMO, AA10, Actinomycetes, Chitin, Cellulose, Xylan

## Abstract

**Background:**

Lytic polysaccharide monooxygenases (LPMOs) opened a new horizon for biomass deconstruction. They use a redox mechanism not yet fully understood and the range of substrates initially envisaged to be the crystalline polysaccharides is steadily expanding to non-crystalline ones.

**Results:**

The enzyme *Kp*LPMO10A from the actinomycete *Kitasatospora papulosa* was cloned and overexpressed in *Escherichia coli* cells in the functional form with native N-terminal. The enzyme can release oxidized species from chitin (C1-type oxidation) and cellulose (C1/C4-type oxidation) similarly to other AA10 members from clade II (subclade A). Interestingly, *Kp*LPMO10A also cleaves isolated xylan (not complexed with cellulose, C4-type oxidation), a rare activity among LPMOs not described yet for the AA10 family. The synergistic effect of *Kp*LPMO10A with Celluclast^®^ and an endo-β-1,4-xylanase also supports this finding. The crystallographic elucidation of *Kp*LPMO10A at 1.6 Å resolution along with extensive structural analyses did not indicate any evident difference with other characterized AA10 LPMOs at the catalytic interface, tempting us to suggest that these enzymes might also be active on xylan or that the ability to attack both crystalline and non-crystalline substrates involves yet obscure mechanisms of substrate recognition and binding.

**Conclusions:**

This work expands the spectrum of substrates recognized by AA10 family, opening a new perspective for the understanding of the synergistic effect of these enzymes with canonical glycoside hydrolases to deconstruct ligno(hemi)cellulosic biomass.

**Electronic supplementary material:**

The online version of this article (10.1186/s13068-019-1449-0) contains supplementary material, which is available to authorized users.

## Background

Lytic polysaccharide monooxygenases (LPMOs) employ an oxidative step to cleave polysaccharides not yet fully understood at the atomic level [1, 2]. Given their flat surfaces, LPMOs are able to access the crystalline portion of polysaccharides releasing new ends that can elicit the activity of canonical enzymes, promoting a boosting on sugar release [3].

LPMOs are classified as auxiliary activities (AAs) and are now distributed on families AA9, 10, 11, 13, 14, 15 on CAZy database (http://www.cazy.org) and, more recently, AA16 [4]. AA10, initially classified as chitin-binding protein 21 (CBP21) or carbohydrate-binding module 33 (CBM33) were first described in *Serratia marcescens* [5] and are widely distributed in bacteria, Actinomycetes being a promising source of these enzymes [6]. Beyond chitin, some AA10 s are also active on cellulose [7].

The fungal counterparts of AA10 enzymes, grouped in the family AA9, recognize cellulose, cello-oligosaccharides [8] and hemicellulose, mainly xyloglucan, which is a polysaccharide closely connected to cellulose [9, 10]. However, the literature is scarce concerning the activity of LPMOs on the main component of hemicellulose, xylan. *Mt*LPMO9A, an AA9 isolated from *Myceliophthora thermophila* C1, releases oxidized xylo-oligosaccharides only when xylan is bound to cellulose [11]. In a similar way, the first component of AA14 family, *Pc*AA14B, from *Pycnoporus coccineus*, only recognizes xylan when interacting with cellulose chains [12]. The only LPMO reported to act on isolated xylan is *Ls*AA9A, from the Basidiomycete *Lentinus similis* [13]. Beyond xylan, *Ls*AA9A is also active on cellulose, xyloglucan, mixed-linkage glucan and glucomannan. Structural analysis of *Ls*AA9A bound to xylo-oligosaccharides suggests other mechanisms of interaction during the catalysis than that observed for cello-oligosaccharides; however, it is unclear for other LPMO families.

The activity of LPMOs on a plethora of substrates is desirable especially when added to enzyme cocktails aiming at the deconstruction of ligno(hemi)cellulosic biomass to simple sugars that can be converted into green chemicals and fuels [14].

In this work, we investigated the functional and structural properties of a LPMO from the actinomycete *Kitasatospora papulosa*, *Kp*LPMO10A. Beyond the already described activities on chitin and cellulose (PASC and Avicel^®^), typical of AA10 from the clade II (subclade A), *Kp*LPMO10A was shown to be active on xylan. This finding expands the range of substrates recognized by this family and depicts a new perspective of the synergistic action of these enzymes with canonical CAZymes to enhance ligno(hemi)cellulosic biomass deconstruction, beyond breaking down glycosidic bonds at crystalline patches in cellulose.

## Results

### *Kp*LPMO10A is a putative chitin/cellulose-oxidizing enzyme from AA10 family

*KpLPMO10A*, a putative LPMO-coding gene (558 base pairs subtracting those spanning the signal peptide) was isolated from the total DNA of *K. papulosa* (Additional file 1: Table S1) and cloned into pET-22b(+) vector downstream of the *pelB* signal peptide to permit the correct processing of the catalytic N-terminal histidine. *Kp*LPMO10A is a non-modular enzyme with 186 amino acids and a predicted molecular mass and isoelectric point (pI) of 20.8 kDa and 4.1, respectively. *Kp*LPMO10A was overexpressed in *Escherichia coli* cells and purified to homogeneity by Ni–NTA metal affinity and size-exclusion chromatography, with a yield of 0.75 mg per liter of culture.

**Fig. 1:**
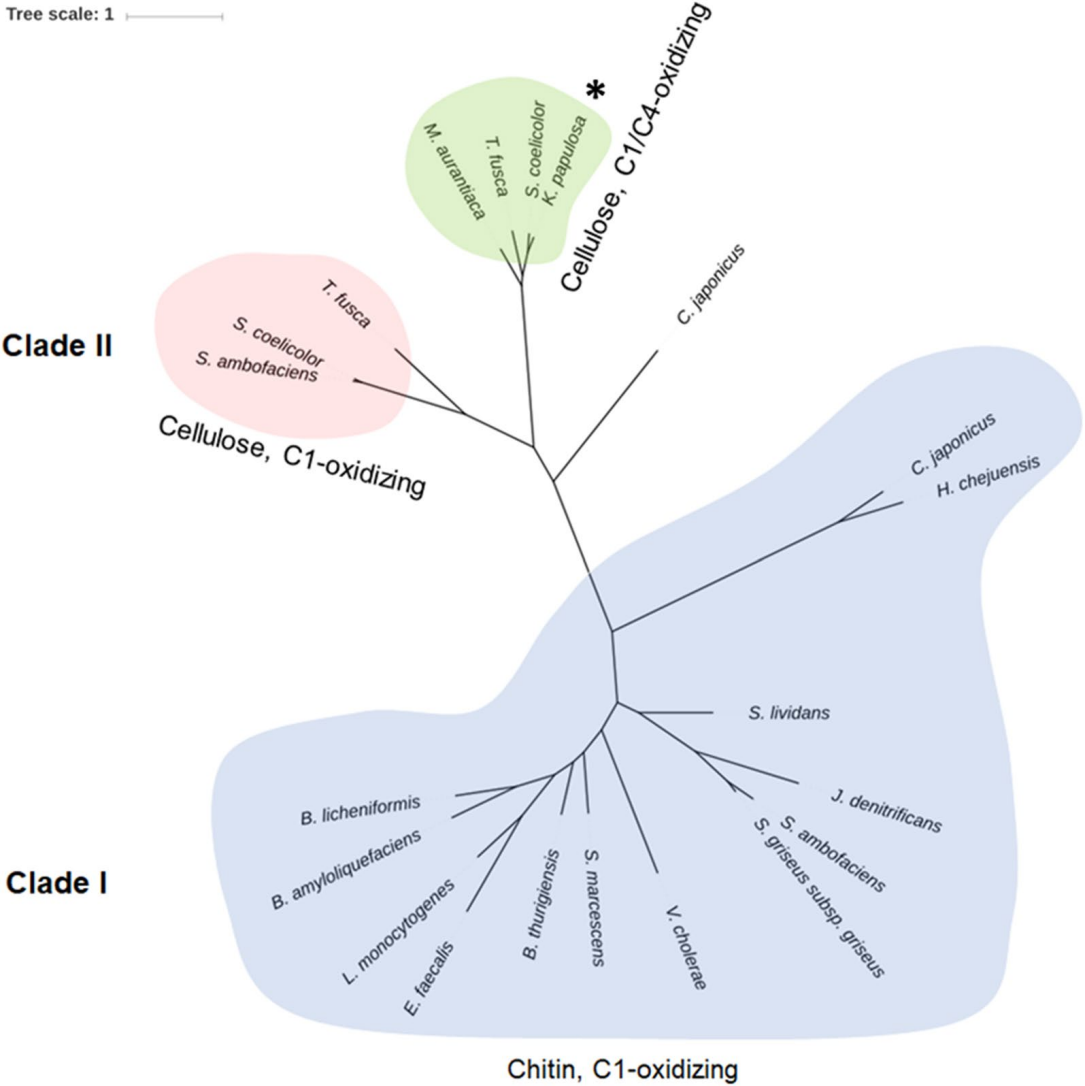
Phylogenetic tree of characterized AA10 proteins available on CAZy database. *Bacillus amyloliquefaciens* (NCBI access code CBI42985.1), *Bacillus licheniformis* (AAU39477.1), *Bacillus thuringiensis* (AJP62637.1), *Cellvibrio japonicus* (ACE83992.1), *C. japonicus* (ACE84760.1), *Enterococcus faecalis* (AAO80225.1), *Hahella chejuensis* (ABC27701.1), *Jonesia denitrificans* (ACV09037.1), *Listeria monocytogenes* (CAD00545.1), *Serratia marcescens* (AAU88202.1), *Streptomyces ambofaciens* (CAJ89556.1), *S. ambofaciens* (CAJ90160.1), *Streptomyces coelicolor* (CAB61160.1), *S. coelicolor* (CAB61600.1), *Streptomyces griseus subsp. griseus* (BAG23684.1), *Streptomyces lividans* (EOY47895.1), *Thermobifida fusca* (AAZ55306.1), *T. fusca* (AAZ55700.1), *Micromonospora aurantiaca* (ADL45185.1), *Vibrio cholerae* (AAF96709.1) and *K. papulosa* (highlighted with an asterisk). The fusolin from *A. cuprea* entomopoxvirus (BAA25629.1) was excluded from this analysis. Clade I, exclusively chitin-oxidizing enzymes; Clade II, chitin/cellulose-oxidizing enzymes (C1 and C1/C4)

The phylogenetic analysis of all characterized auxiliary activity 10 (AA10) proteins available on CAZy database (access October 2018), except the fusolin from *Anomala cuprea* entomopoxvirus, classified the sequences in two clades as predicted by [15] (Fig. 1). Clade I comprises those enzymes exclusively active on chitin, whereas the clade II (subclade A) encompasses LPMOs with the ability to recognize both chitin and cellulose.

*Kp*LPMO10A clustered with LPMOs protein sequences from other Actinomycetes such as *Streptomyces coelicolor Sc*LPMO10B (86% primary sequence identity with *Kp*LPMO10A, [7]), *Thermobifida fusca* E7 (69% primary sequence identity, [16]) and *Micromonospora aurantiaca* Micau_1230 (65% primary sequence identity, [17]). All these LPMOs oxidize the carbon 1 (C1) and/or carbon 4 (C4) of glucose in cellulose chains, suggesting that *Kp*LPMO10A presents the same regioselectivity. These enzymes are also active on chitin (only C1 oxidation). Compared to chitin/cellulose strictly C1-oxidizing enzymes, *Kp*LPMO10A presents a primary sequence identity of 24% (*Serratia marcescens* CBP21) and 31% (*S. coelicolor* CelS2), respectively.

### *Kp*LPMO10A oxidizes cellulose (C1/C4) and chitin (C1)

*Kp*LPMO10A was predicted as a cellulose/chitin-oxidizing protein by our phylogenetic analysis. Preliminary substrate-binding assays monitored by SDS-PAGE (Additional file 2: Figure S1) showed that *Kp*LPMO10A binds preferably to cellulosic substrates (Avicel^®^ and PASC, phosphoric acid-swollen cellulose) than α-chitin considering the proportion of enzyme that remained bound to the substrate (insoluble fraction).

**Fig. 2:**
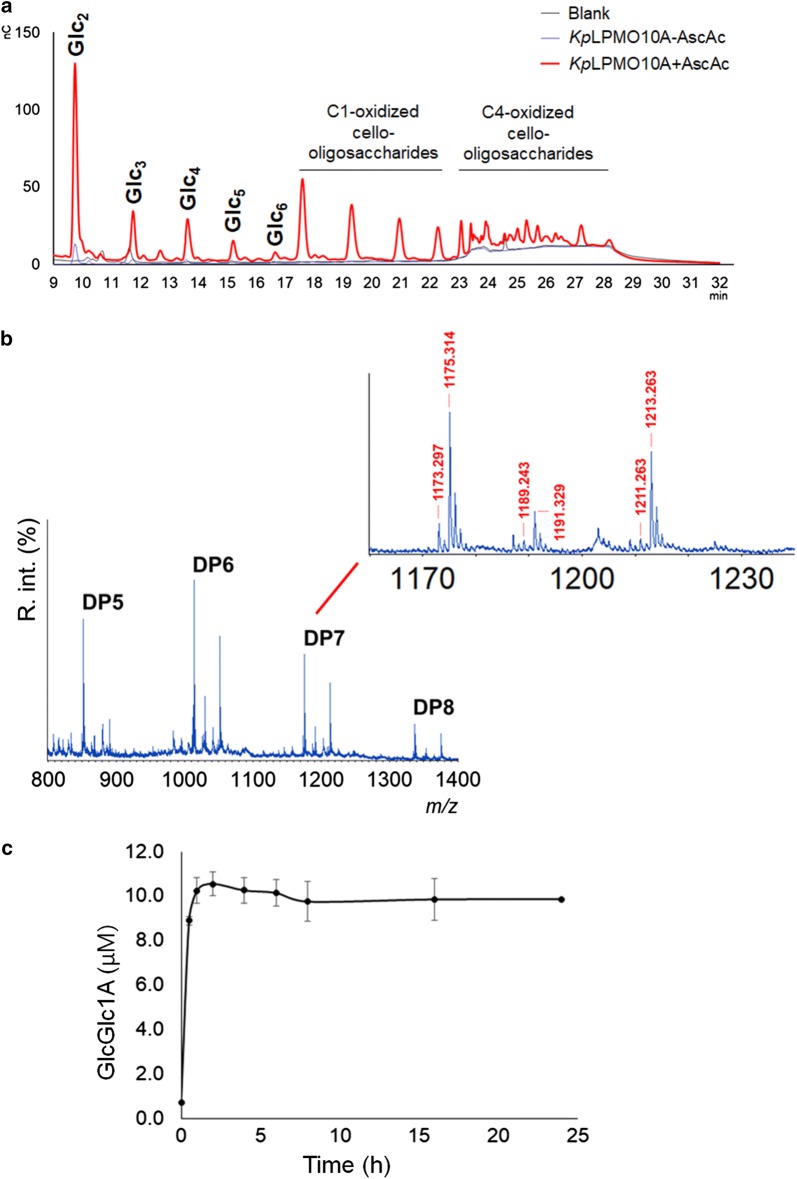
HPAEC-PAD chromatogram showing oligosaccharides released by *Kp*LPMO10A from PASC after 16 h at 37 °C in the presence or absence of ascorbic acid. Blank reactions were carried out with buffer, PASC and ascorbic acid. Peaks of native (Glc_2_–Glc_6_) and oxidized cello-oligosaccharides were assigned based on standard cello-oligosaccharides (Megazyme Inc.) and [57], respectively (**a**). MALDI-TOF MS data confirmed the release of native and C1/C4-oxidized oligosaccharides from PASC. The inset shows DP7, *m/z* 1175.314 (native); lactone or ketoaldose, *m/z* 1173.297 (− 2 Da); aldonic acid or gemdiol, *m/z* 1191.329 (+ 16 Da) and di-sodiated adduct of aldonic acid, *m/z* 1213.263 (+ 38 Da). Additionally, *m/z* 1189.243 (+ 14 Da) and 1211.263 (+ 36 Da) correspond to the double-oxidized products and their sodium adducts, respectively (**b**). Release of cellobionic acid (GlcGlc1A) from PASC over 24 h at 37 °C/850 rpm (**c**)

HPAEC-PAD chromatograms show the release of native (Glc_2_ to Glc_6_) and C1-oxidized cello-oligosaccharides (17–23 min) from PASC after treatment with *Kp*LPMO10A during 16 h/37 °C (Fig. 2a). Native cello-oligosaccharides appearing in the HPAEC-PAD chromatogram are partly due to on-column degradation of C4-oxidized species, in addition to the native ones naturally formed in the reaction [18]. Evidences of C4-oxidized products were observed in elution times from 23 min (Fig. 2a). In the absence of a reducing agent (ascorbic acid, AscAc), *Kp*LPMO10A is not active against PASC as observed upon its addition (Fig. 2a). Reactions with Avicel^®^ resulted in the same product profile, but peaks were less intense than that observed for PASC, which is typical for LPMOs (Additional file 3: Figure S2A).

Unmodified cello-oligosaccharides with degree of polymerization (DP) 5–8 and their C1 and/or C4-oxidized counterparts from PASC were detected by MALDI-TOF MS (Fig. 2b). The inset shows the mono-sodiated unoxidized form of celloheptaose (*m/z* 1175.314), mono-sodiated lactone or ketoaldose (*m/z* 1173.297), and mono- and di-sodiated adducts of C1-aldonic acids (*m/z* 1191.329 and 1213.263, respectively). In addition, the *m/z* 1189.243 and 1211.263 correspond to the double-oxidized (C1 and C4) products and their sodium adducts, respectively. This profile unequivocally indicates that *Kp*LPMO10A releases C1 and/or C4-oxidized products. A similar profile was found for *Sc*LPMO10B [7].

The conversion of PASC into cellobionic acid (GlcGlc1A) was quantified over 24 h (Fig. 2c). *Kp*LPMO10A remains active for about 2–4 h, releasing 80% of the total GlcGlc1A within the first 30 min. Around 0.4% of PASC was converted into GlcGlc1A in the conditions assayed.

Activity of *Kp*LPMO10A was not observed when the cello-oligosaccharides Glc_5_ and Glc_6_ were used as substrates.

Bacterial LPMOs (AA10) were first described as chitin-binding proteins [1]. Incubation of α-chitin with *Kp*LPMO10A on the same conditions adopted for PASC promoted the release of chito-oligosaccharides mainly from DP3 to 8. Additional file 4: Figure S3A highlights the mono-sodiated DP5 (*m/z* 1056.281) as well as its mono-sodiated lactone or ketoaldose (*m/z* 1054.276), the mono-sodiated aldonic acid or gemdiol (*m/z* 1072.300) and the di-sodiated adduct of aldonic acid (*m/z* 1094.300), indicative of C1-oxidation, thus confirming the oxidative cleavage of α-chitin by *Kp*LPMO10A.

Experiments with colloidal chitin released chito-oligosaccharides up to DP9 (Additional file 4: Figure S3B). Overall, the intensity of oxidized peaks was higher for this substrate than that observed for non-treated α-chitin. In addition, DP6 was the most intense peak, as observed for PASC treatment in contrast with the ladder pattern observed for α-chitin. Additional file 4: Figure S3B (inset) also shows the pentamer released from colloidal chitin.

### *Kp*LPMO10A boosts sugar release from Avicel^®^, Filter Paper and pretreated sugarcane bagasse

**Fig. 3:**
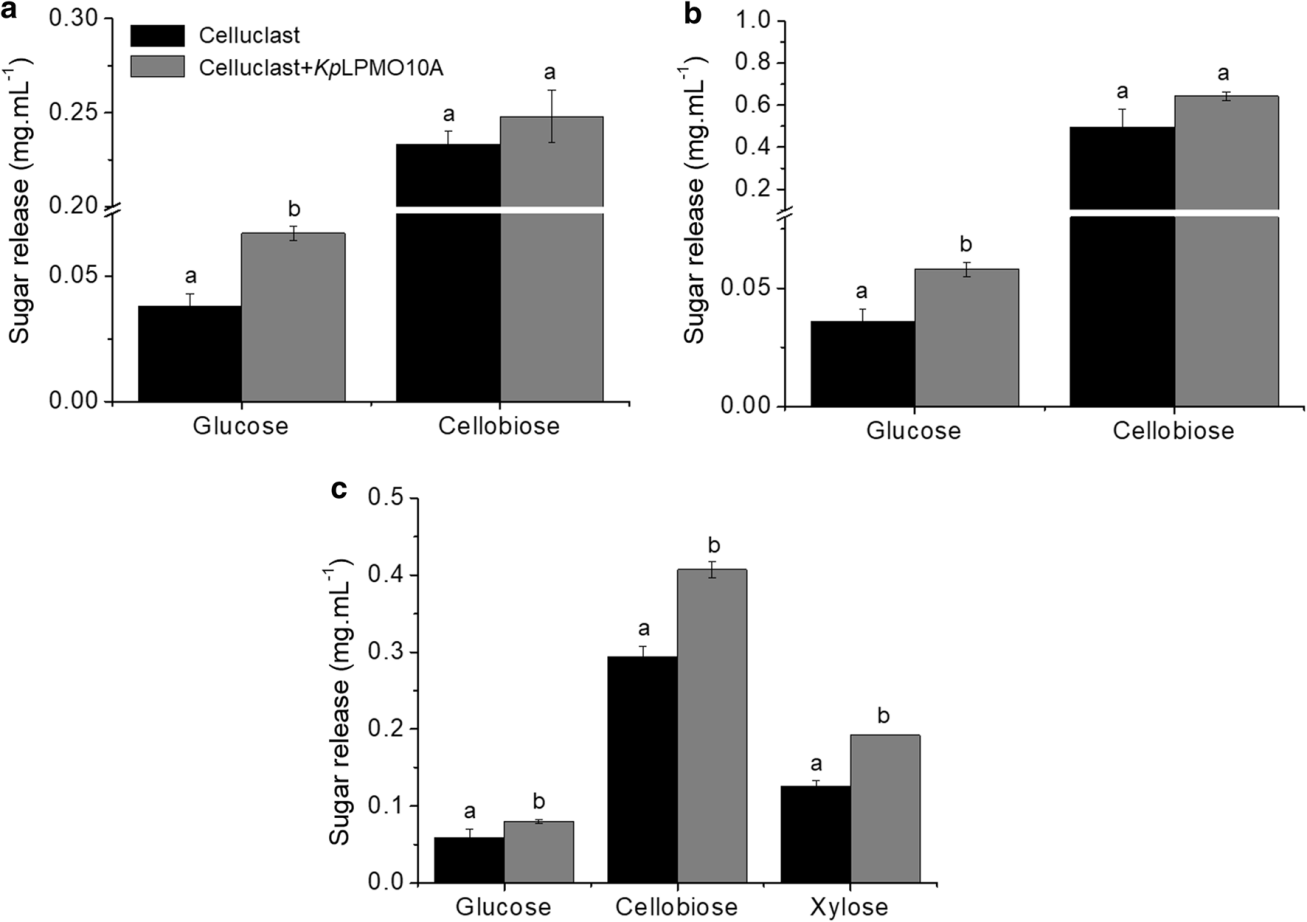
Synergy of *Kp*LPMO10A and Celluclast^®^. Release of sugars from Avicel^®^ (**a**), Filter Paper (**b**) and pretreated sugarcane bagasse (**c**). The experiments were carried out at 50 °C for 24 (Avicel^®^ and Filter Paper) and 72 h (sugarcane bagasse). Control reactions were done with only Celluclast^®^. Groups of bars with the same letter mean no statistical difference at 0.05 significance by Tukey test

Given the activity of *Kp*LPMO10A on cellulosic substrates, we investigated its synergy with Celluclast^®^ to deconstruct Avicel^®^, Filter Paper and alkaline-treated sugarcane bagasse (Fig. 3). The experiments were carried out at 50 °C and pH 5.0 to promote the activity of Celluclast^®^. The boosting on glucose release reached 76 and 61% for Avicel^®^ and Filter Paper (Fig. 3a, b), respectively. When alkaline-treated sugarcane bagasse was used as substrate, a total of 25 and 38% more glucose and cellobiose, respectively, were measured upon the addition of *Kp*LPMO10A. Interestingly, the concentration of xylose was also higher in this condition (52%), suggesting synergy with enzymes other than cellulases eventually present in the cocktail. Assays with sugarcane bagasse were not added with ascorbic acid given the potential role of lignin components as electron donors.

### Activity of *Kp*LPMO10A on xylan expands the substrate spectrum recognized by bacterial LPMOs

Given the boost on xylose release observed in the synergy experiments, we evaluated whether *Kp*LPMO10A is able to oxidatively cleave hemicellulosic compounds. Enzymatic reactions with 2 mg/mL xyloglucan, guar galactomannan and konjac glucomannan resulted in HPAEC-PAD profiles identical to the control ones carried out in the absence of *Kp*LPMO10A (Additional file 3: Figure S2B–D). These reactions were also evaluated by MALDI-TOF MS and no products were observed (data not shown).

**Fig. 4:**
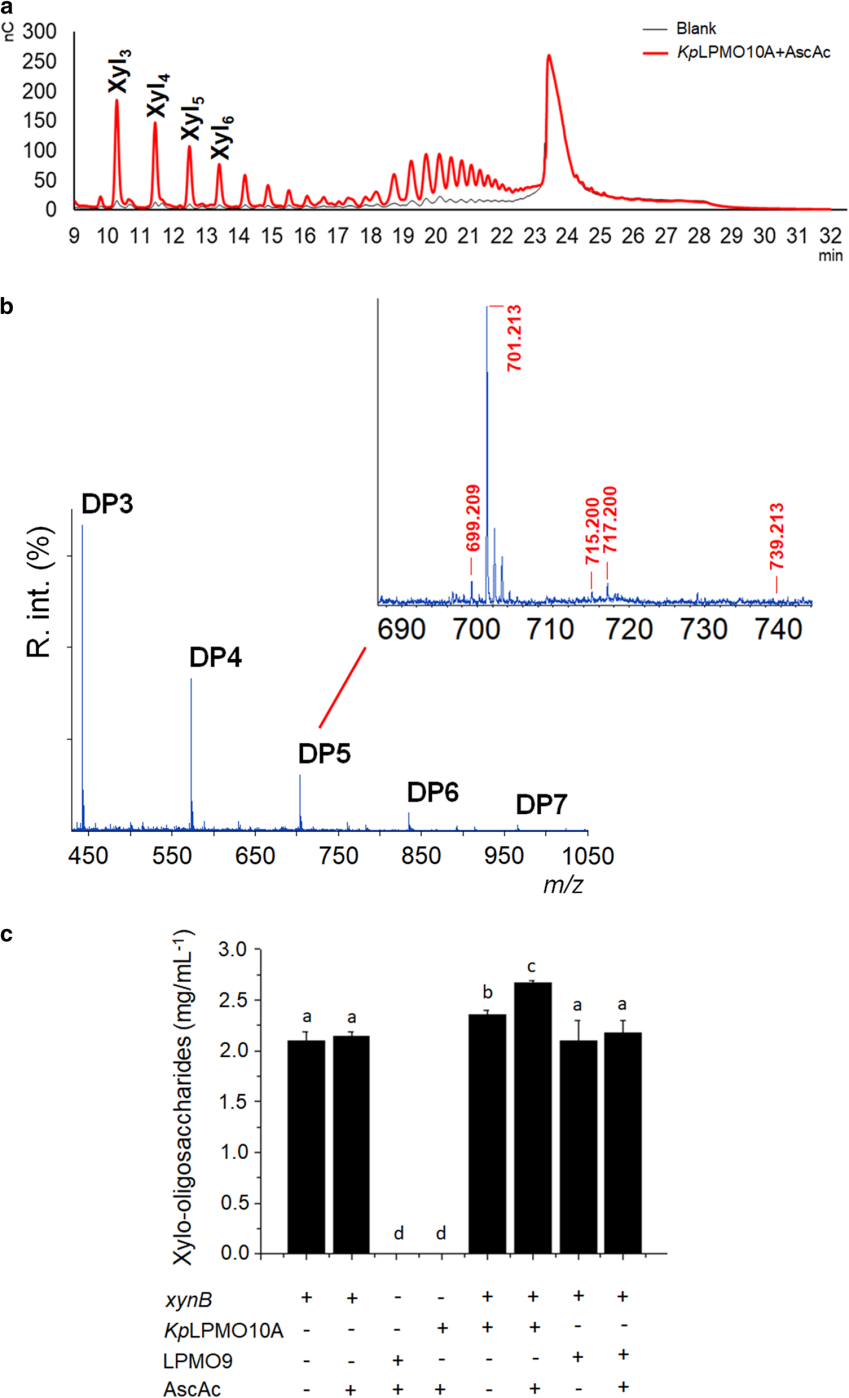
HPAEC-PAD chromatogram showing oligosaccharides released by *Kp*LPMO10A from beechwood xylan after 16 h at 37 °C. Blank reactions were carried out with buffer, xylan and ascorbic acid. Peaks of native (Xyl_3_–Xyl_6_) xylo-oligosaccharides were assigned based on a standard (Megazyme Inc.) (**a**). MALDI-TOF MS data. The inset shows the sodiated forms of DP5, *m/z* 701.213 (native); lactone or ketoaldose, *m/z* 699.209 (− 2 Da) and aldonic acid or gemdiol, *m/z* 717.200 (+ 16 Da). No peaks related to the sodium adduct of the sodium salt of aldonic acid (*m/z* 739.213) were observed. *m/z* 715.200 (+ 14 Da) was assumed as the potassium adduct of native DP5 (and not an indicative of double oxidation) given that no C1-oxidized species are produced by *Kp*LPMO10A (see Additional file 6: Figure S5) (**b**). Synergy of *Kp*LPMO10A and LPMO9 (an strictly cellulose-oxidizing LPMO) with the endo-β-1,4-xylanase *xyn*B/XAC4254 (**c**). Synergy experiments were carried out with beechwood xylan as substrate at 37 °C during 24 h/1000 rpm. Control reactions were done with only *xyn*B or *Kp*LPMO10A or LPMO9. Bars with different letters mean statistical difference at 0.05 significance by Tukey test

Xylan is a polymer of β-(1→4)-d-xylosyl residues that resembles the β-(1→4)-d-glucan found in cellulose [19]. Incubation of *Kp*LPMO10A with xylan in the same conditions adopted for cellulose and chitin gave rise to several products and the corresponding peaks were eluted from 10 to 23 min according to HPAEC-PAD profile (Fig. 4a). Peaks from 10 to 14 min were assigned as Xyl_3_ to Xyl_6_.

Control reactions using copper sulfate (CuSO_4_) at the same concentration as *Kp*LPMO10A (4.4 μM) along with hydrogen peroxide (0, 10, 25, 50, 75 and 100 μM), ascorbate (1 mM), buffer and xylan from beechwood (2 mg/mL) were performed to ensure that the possible oxidized products did not originate from Fenton reactions. No peaks concerning oxidized species were detected by HPAEC-PAD using these reactants (Additional file 5: Figure S4).

The occurrence of oxidized products was evaluated by MALDI-TOF MS (Fig. 4b). Xylo-oligosaccharides from DP3 to DP7 were produced by *Kp*LPMO10A. Peaks related to the mono-sodiated lactone/ketone (*m/z* 699.209) and mono-sodiated gemdiol/aldonic acid (*m/z* 717.200) species are highlighted for the pentamer. Additionally, the sodium adduct of the sodium salt of aldonic acid (*m/z* 739.213), determinant of C1 oxidation, is not visible.

The absence of C1-oxidized products was further confirmed by comparing the HPAEC-PAD profile of xylan/*Kp*LPMO10A reactions with C1-oxidized xylo-oligosaccharides produced upon the enzymatic treatment of Xyl_2_–Xyl_5_ with cellobiose dehydrogenase (CDH) (Additional file 6: Figure S5). No peaks corresponding to C1-oxidized species were found, suggesting that the oxidative cleavage of xylan by *Kp*LPMO10A occurs exclusively at C4.

*Kp*LPMO10A activity on isolated xylan was also demonstrated by synergy assays with the endo-β-1,4-xylanase *xyn*B (GH11, [20]). Experiments were performed using xylan from beechwood as substrate and the release of xylo-oligosaccharides was measured after 24 h reaction. Reactions with *Kp*LPMO10A (ascorbic acid added) released 25% more oligosaccharides compared to the control experiment (Fig. 4c). Analyzing the released xylo-oligosaccharides separately, it was observed that the increase in Xyl_2_ and Xyl_3_ reached 23 and 24%, respectively, Xyl_2_ being the main product. Results regarding Xyl, Xyl_4_, Xyl_5_ and Xyl_6_ did not show statistically significant differences (data not shown).

The addition of the strictly cellulose-oxidizing LPMO from fungal origin “LPMO9” (Corrêa et al. to be submitted) to *xyn*B did not increase the conversion of xylan into xylo-oligosaccharides, thus, validating the xylan-oxidizing activity of *Kp*LPMO10A (Fig. 4c).

Xyl_5_ and Xyl_6_ were also evaluated as substrates for *Kp*LPMO10A, but no products were recorded.

### *Kp*LPMO10A catalytic interface is indistinguishable from other clade II (subclade A) AA10 LPMOs

**Fig. 5:**
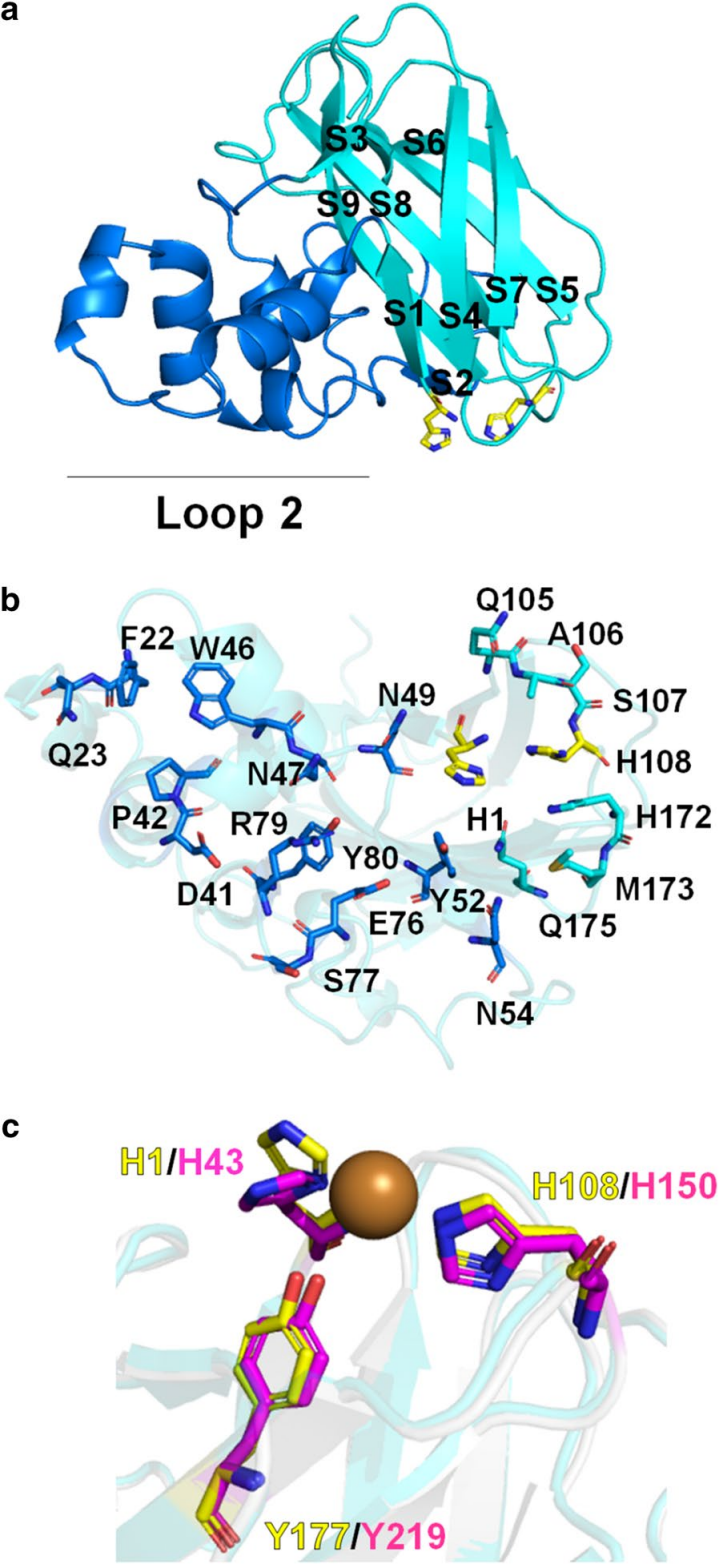
*Kp*LPMO10A structure showing the fibronectin/immunoglobulin-like β-sandwich fold typical of LPMOs. β-sheets are highlighted in light blue and loop 2 in blue (**a**). The substrate-binding residues were annotated following [17] (**b**). Superimposing of *Kp*LPMO10 with *Sc*LPMO10B (PDB code: 4OY6) showing the residues related to copper coordination (**c**). S, strand

To understand the structural basis of *Kp*LPMO10A activity on both crystalline and non-crystalline substrates, we have solved its crystal structure at 1.6 Å resolution (Table 1). As expected, *Kp*LPMO10A displays the fibronectin/immunoglobulin-like β-sandwich fold typical of LPMOs [21] with two β-sheets, the first one comprising three antiparallel strands (S1, S4 and S7) and the later with four parallel strands (S5, S6, S8 and S9). The 85-residue-long motif consisting of short α-helices and loops between the strands S1 and S4 of the antiparallel β-sheet is known as loop 2 (Fig. 5a). This motif forms a significant patch of the substrate binding (Fig. 5b) that is conserved among the characterized chitin and C1/C4-cellulose-oxidizing AA10 s (Additional file 7: Figure S6).

**Table 1:** *Kp*LPMO10A crystal data collection and refinement statistics

	*Kp*LPMO10A
Data collection	
Wavelength (*λ*)	1.458
Space group	P 1 21 1
Cell dimensions	
*a, b, c* (Å)	37.21, 109.57, 42.78
*α*, *β*, *γ* (o)	90, 113.5, 90
Resolution (Å)	24.94–1.60 (1.66–1.60)
*R*_meas_	0.086 (0.861)
*I*/*σI*	8.90 (1.33)
Completeness (%)	96.55 (85.36)
Redundancy	3.2 (2.5)
Refinement	
Resolution (Å)	24.94–1.60 (1.66–1.60)
No. reflections	126,755 (8747)
*R*_work_/*R*_free_	0.196 (0.352)/0.237 (0.417)
No. of atoms	
Protein	3234
Ligand/ion	0
Water	212
Protein residues	379
Ligands	0
B factors (Å^2^)	
Average	24.62
Protein	24.41
Ligand/ions	0
Water	27.68
R.m.s. deviations	
Bond lengths (Å)	0.007
Bond angles (^o^)	0.85
Ramachandran	
Favored (%)	96.8
Allowed (%)	3.2
Outliers (%)	0.00
PDB code	6NDQ

Statistics for the highest resolution shell are shown in parenthesis

No copper occupancy was observed in *Kp*LPMO10A structure, but the superimposition with *Sc*LPMO10B (PDB code: 4OY6) (Fig. 5c) showed that all residues relevant for copper coordination are fully conserved, including the histidines 1 and 108 (brace of histidines, H1 and H108) and the tyrosine 177 (Y177). This aromatic residue is conserved among AA10s and occupies an axial position for copper coordination. Conserved residues neighboring the copper ion center also include Asp104, Ala106, His172 and Gln175. Studies carried out by [17] showed that mutations on residues near the copper center led to a decrease in activity, but did not affect the regioselectivity displayed by C1/C4-oxidizing bacterial LPMOs.

Although the copper was not found in both molecules in the asymmetric unit, thermal shift assays revealed its key role in thermal stability of *Kp*LPMO10A with an increase in melting temperature (*T*_*m*_) of ~ 12 °C and ~ 10 °C at pH 5.0 and 6.0, respectively (Additional file 8: Figure S7). The process was shown to be reversed following the addition of EDTA to copper-saturated *Kp*LPMO10A as also assayed by circular dichroism spectroscopy (data not shown).

The catalytic interface of *Kp*LPMO10A was compared in detail with other structurally characterized AA10 members from the clade II (subclade A) including *Sc*LPMO10B, Micau_1230 (PDB code: 5OPF) and E7 (PDB code:  5UIZ), which did not reveal any evidence of structural and conformational difference that could be correlated with the ability to oxidize xylan chains. This analysis suggests that closely related members may also be active on xylan, being the clade II (subclade A), a polyspecific subfamily of the AA10 family or that the substrate recognition and binding involve yet elusive mechanisms.

## Discussion

LPMOs are enzymes that have revolutionized the polysaccharide deconstruction field. Recent discoveries such as the activity on hemicellulose [9] and starch [22], alternative electron donors [23–25] and co-substrates [2] point out that our knowledge about these biocatalysts is in its infancy. Further studies are required to shed light on the catalytic cycle steps, substrate selectivity, synergy with other CAZymes and the biological roles on carbon cycle. The expansion on the understanding of these aspects is instrumental for industrial applications.

LPMOs are widespread in nature [26] and bacterial genomes are estimated to have 0–7 LPMO-coding genes [27]. Some actinomycetes have been explored as sources of these enzymes [7, 17, 28]. Among them, *Kitasatospora* sp. is found in soil and harbors genes related to the depolymerization of chitin, cellulose and hemicellulose [29]. *K. papulosa* has five putative chitin-binding coding genes. Our target, *Kp*LPMO10A, shares 29–31% amino acid identity with the endogenous chitin-binding proteins, but higher identity levels with the characterized ones from *S. coelicolor* (86%), *T. fusca* (69%) and *M. aurantiaca* (65%). *Kp*LPMO10A has the typical fold of AA10s and AA9s [30, 31] and given the identity with *Sc*LPMO10B (86%), several *Kp*LPMO10A structural features were discussed by [7].

The prediction of *Kp*LPMO10A as a chitin and C1/C4-cellulose-oxidizing enzyme was confirmed by experimental assays. *Kp*LPMO10A showed a product profile similar to *Sc*LPMO10B, Micau_1230 and E7 acting on PASC [7, 17, 32]. PASC probably adopts the same orientation in the similar binding patch and catalytic center shared by these enzymes. Mutation studies from Micau_1230 showed that the regioselectivity of AA10 relies on the positioning of substrate on the copper site [17], while for AA9 it sounds to be determined by structural features of loop 2, aromatic residues on substrate-binding patch and exposed *N*-glycans [33, 34].

*Kp*LPMO10A releases GlcGlc1A after up to 2 hours of reaction, which resembles the production of C4 oxidized species by *Mt*LPMO9J [35]. For *Pa*LPMOH, the release of GlcGlc1A is time dependent [36].

*Kp*LPMO10A is also active on α-chitin, releasing oxidized chito-oligosaccharides. A preference of AA10s for releasing even-numbered products from chitin has been shown [1, 37, 38]. *Kp*LPMO10A releases preferentially products with lower DPs, as also shown for *Ba*AA10 from *Bacillus amyloliquefaciens* [39]. Curiously, *Sc*LPMO10B did not show activity on this substrate, only β-chitin [7].

Treatment of α-chitin with phosphoric acid (“colloidal chitin”) gives rise to a substrate with low crystallinity, molecular mass, surface area and particle size [40]. *Kp*LPMO10A was also able to release oxidized products from this substrate, with preference for DP6. Beyond this, the DP of products released was extended, in this particular case to 9, which is in accordance with data from [41]. In addition, peaks regarding the oxidized products were higher than those observed for α-chitin.

Synergy assays of *Kp*LPMO10A with Celluclast^®^, an enzyme cocktail containing mainly cellobiohydrolase (CBH) and endo-1,4-β-glucanase (EG) activities [42], promoted an increase in glucose and cellobiose release from Avicel^®^ and Filter Paper, highlighting the potential application of this enzyme as additive in enzyme cocktails. Some examples of sugar release boosting by LPMOs are highlighted in [14]. We also observed higher titers of xylose, in addition to glucose and cellobiose, when sugarcane bagasse was employed as substrate. Beyond CBHs and EGs, Celluclast^®^ contains a range of other activities, for example, xylanases [43]. Then, we hypothesized that *Kp*LPMO10A could act also in hemicellulosic components.

The activity on hemicellulosic components such as xyloglucan and glucomannan, for example, is known for AA9s [9, 44, 45]. *Kp*LPMO10A does not cleave polysaccharides with β-(1→4) glycosidic bonds with α-(1→6) xylosyl branches (xyloglucan), β-(1→4) mannan and randomly distributed glucose (glucomannan) and β-(1→4) mannan with α-(1→6) galactosyl branches (guar galactomannan). However, *Kp*LPMO10A acts on β-(1→4) xylan.

Activity on isolated xylan (not complexed to cellulose), as shown by *Kp*LPMO10A, is a rare event for LPMOs. The only record is found for *Ls*AA9A, from *L. similis,* which oxidizes xylan and xylo-oligosaccharides (Xyl_6_) at C1/C4 and C4, respectively [13]. Other two works show that the release of oxidized products from xylan by MtLPMO9A (C1/C4 oxidation) and *Pc*AA14B (C1 oxidation) is only possible when this substrate is complexed with cellulose [11, 12]. *Pc*AA14B is the first member of AA14 family, comprising exclusively lytic xylan oxidases. In contrast to that observed for *Pc*AA14B and *Mt*LPMO9A, which oxidize xylan at C1 and C1/C4, respectively, *Kp*LPMO10A releases C4-oxidized species, which was confirmed by HPAEC-PAD analysis.

Comparison of *Kp*LPMO10A, *Ls*AA9A (5NLO) and *Pc*AA14B (5NO7) structures did not show any clue about the activity/regioselectivity on xylan (Additional file 9: Figure S8). *Ls*AA9A is the only LPMO structure complexed with substrates (cello and xylo-oligosaccharides) available in the Protein Data Bank. *Ls*AA9A presents a less pronounced binding surface and a protuberance near the subsites + 1 and + 2 [46], while *Pc*AA14B has a clamp formed by two surface loops [12]. These data suggest different structural determinants for xylan specificity in LPMOs. Unfortunately, the structure of *Mt*LPMO9A, the other LPMO that cleaves xylan, is not available.

This work is the first to describe the activity of an AA10 member on xylan. Structural comparisons with other AA10, such as *Sc*LPMO10B, Micau_1230 and E7 indicate no significant differences that would explain the ability of *Kp*LPMO10A to act on xylan, which tempted us to suggest that these homologues might also exhibit this similar feature or yet obscure mechanisms of substrate interaction are involved.

The activity on xylan is also corroborated by synergy assays with Celluclast^®^ and the endoxylanase *xyn*B. *Kp*LPMO10A promoted a higher overall sugar release from biomass when compared to an exclusively cellulose-oxidizing AA9 (LPMO9) also studied by our group and the activity on hemicellulose probably facilitates the accessibility to cellulose chains.

## Conclusions

In this work, we describe the cloning, overexpression, functional and structural characterization of a new bacterial LPMO (named as *Kp*LPMO10A), which is active on xylan, beyond the classical activity on chitin and cellulose (PASC/Avicel^®^). *Kp*LPMO10A is the first bacterial LPMO to exhibit such activity on xylan and only very few fungal LPMOs are able to attack this polysaccharide. Moreover, *Kp*LPMO10A shows a regioselectivity for xylan (C4) distinct to that observed for cellulosic substrates (C1/C4), which could serve as an excellent model to address mechanistic questions regarding regioselectivity. The discovery of new activities in already known families opens new horizons regarding the mechanistic of LPMOs, its role on carbon cycle and its suitability for new industrial applications.

## Methods

### Isolation and cloning of *Kp*LPMO10A

The genomic DNA of *K. papulosa* (DSM41643) was extracted according to [47] with modifications. The gene *KpLPMO10A* was amplified by polymerase chain reaction (PCR) using the primers KpF and KpR (Additional file 1: Table S1). The reaction contained 0.25 μL of Phusion DNA Polymerase (New England Biolabs), 5 μL of 5X buffer, 0.25 μL of 50 mM MgCl_2_, 1 μL of 10 mM dNTPs, 2 μL of 5 μmol each primer and 12.5 μL of water. PCR conditions were as follows: initial denaturation at 98 °C for 30 s, 35 denaturation cycles at 98 °C for 10 s, annealing at 61 °C for 30 s, extension at 72 °C for 30 s and a final extension at 72 °C for 10 min.

The amplicon (558 base pairs) was purified using QIAquick gel extraction kit (Qiagen) and employed as a template for a second round of PCR using the primers KpFpET22b and KpRpET22b (Additional file 1: Table S1). KpFpET22b and KpRpET22b were designed to add a homology sequence to vector pET-22b(+). The same reaction conditions highlighted above were adopted in this step.

The purified product was inserted into pET-22b(+) immediately after *pel*B coding sequence to ensure the histidine as the first amino acid and the His_6_-tag at C-terminus by Gibson Assembly methodology [48]. The reaction products were transformed into thermocompetent *Escherichia coli* DH5α cells. pET-22b(+)-*Kp*LPMO10A was purified using QIAprep Miniprep System (Qiagen) and was sequenced to confirm the correct integration of *KpLPMO10A* in pET-22b(+).

### Overexpression and purification

Thermocompetent cells of *E. coli* SHuffle^®^ transformed with pET-22b(+)-*KpLPMO10A* were grown on Terrific Broth (TB) at 30 °C/250 rpm until it reached an optical density of 0.6. After induction with 0.5 mM isopropyl β-d-1-thiogalactopyranoside (IPTG), cells were maintained overnight at 16 °C. The cells had the periplasmic fraction extracted by osmotic shock protocol [49]. The supernatant was loaded onto a His-Trap column 5 mL (GE Healthcare) equilibrated with buffer A (0.02 M potassium phosphate pH 7.4, 0.5 M NaCl and 0.05 M imidazol). Bound proteins were eluted following a gradient of Buffer B (0.02 M potassium phosphate pH 7.4, 0.5 M NaCl and 0.5 M imidazol). Fractions containing *Kp*LPMO10A were pooled together, concentrated with Vivaspin (cutoff 10 kDa, Sartorius) and applied onto HiLoad Superdex G-75 16/60 (GE Healthcare) previously equilibrated with 0.02 M potassium phosphate pH 7.4 and 0.15 M NaCl. Elution was achieved at a flow rate of 1 mL/min using the same buffer. All fractions were evaluated by SDS-PAGE [50] and those containing the enzyme were pooled together, concentrated and employed for further assays. All purification steps were performed in Äkta Purifier System (GE Healthcare).

Purified enzyme was treated with CuSO_4_ (3:1 protein) overnight at 4 °C. The copper excess was removed with a desalting column Sephadex G-25 PD-10 (GE Healthcare). Protein concentration was estimated (mg/mL) using absorbance spectroscopy at 280 nm in a Nanodrop spectrometer (Thermo Scientific). The molecular mass (kDa) and molar extinction coefficient (ε/1000) were extracted from the sequence using the ExPAsy-ProtParam Tool (https://web.expasy.org/protparam/).

### Crystallization, data collection, structure determination and refinement

*Kp*LPMO10A was crystallized by the sitting-drop vapor-diffusion method at 18 °C using a protein concentration of 6 mg/mL. Crystals were obtained in 0.1 M MMT buffer, pH 4.0 and 25% (v/v) polyethylene glycol (PEG) 1500. Diffraction data were collected on the beamline MX2 at the Synchrotron Light Source Laboratory, LNLS, Campinas, São Paulo, Brazil. A dataset corresponding to 180º was obtained using the fine-slicing strategy (0.1 per image). The wavelength was set to 1.46 Å and a PILATUS2 M detector was used to record the reflections (Dectris, Baden-Dattwil, Switzerland). Data were integrated and scaled using the XDS package [51]. *Kp*LPMO10A was solved by molecular replacement using the crystal structure of the LPMO from *Streptomyces* coelicolor (PDB code 4OY6) [7]; sequence identity of 86%] using the program phaser [52] as a module of PHENIX package [53].

The final model was obtained after several manual building cycles using Coot [54] along with restrained refinement using REFMAC5 [55]. The atomic coordinates and diffraction data were deposited on Protein Data Bank (https://www.pdb.org/) under the code 6NDQ. Molecular images were produced with PyMOL software (Schrödinger).

### Differential scanning fluorimetry (DSF)

The stability of *Kp*LPMO10A in the presence of copper was evaluated by differential scanning fluorimetry (DSF) in a ViiA 7 Real-Time PCR System equipment (Thermo Fischer Scientific). The buffers 0.05 M sodium acetate, pH 5.0, or 0.05 M potassium phosphate, pH 6.0, were mixed with 15 μL (1.3 mg/mL) of copper-treated or -non-treated *Kp*LPMO10A and 15 μL of Sypro Orange (66-fold diluted from a concentrated solution). The fluorescence was measured following the increase in temperature from 30 to 70 °C. The melting temperature (*T*_*m*_) was obtained by fitting a sigmoidal curve in the Origin Software (Origin Lab).

### Binding assays

Binding assays were performed as recommended by [56]. Phosphoric acid-swollen cellulose (PASC) 1%, Avicel^®^ 5% and α-chitin from shrimp shells 1% were incubated with 80 μg of *Kp*LPMO10A and 0.05 M potassium phosphate buffer, pH 6.0, in a total volume of 200 μL for 1 h on ice and gentle mixing. Additionally, reactions were carried out at 37 °C/850 rpm during 16 h. Ten millimolar of EDTA was added to prevent catalytic activity. After incubation, the samples were centrifuged at 10,000*g* for 5 min and the supernatants were considered as the soluble fraction. The pellet was washed twice with buffer obeying the same volume of the initial reaction. The remaining pellet was treated with SDS-PAGE without dye to dissociate the bound proteins (insoluble fraction). All reactions were evaluated by SDS-PAGE.

### Enzyme assays

All reagents used for enzymatic reactions were purchased from Sigma-Aldrich (chitin from shrimp shells and Avicel^®^) or Megazyme (xylan from beechwood, xyloglucan, glucomannan, guar galactomannan, Glc_2_–Glc_6_ and Xyl_2_–Xyl_6_). All used substrates are described in Additional File 10: Table S2. PASC and colloidal chitin were obtained upon treatment of Avicel^®^ and α-chitin from shrimp shells, respectively, with phosphoric acid [40, 57]. PASC (1 mg/mL), Avicel^®^ (1 mg/mL), α- and colloidal chitin (5 mg/mL), xylan from beechwood (2 mg/mL), xyloglucan (2 mg/mL), glucomannan (2 mg/mL) and guar galactomannan (2 mg/mL) were mixed with 4.4 μM *Kp*LPMO10A, 0.05 M sodium phosphate buffer, pH 6.0, and 1 mM ascorbic acid in a total volume of 300 μL. Reactions with the oligosaccharides Glc_5_, Glc_6_, Xyl_5_ and Xyl_6_ (50 μM) were done with 0.02 M sodium phosphate buffer, pH 6.0. Unless otherwise stated, all reactions were carried out at 37 °C/850 rpm for 16 h. After this, the samples were boiled for 10 min and centrifuged at 12,000*g* to separate the soluble and insoluble fractions. The release of cellobionic acid from PASC by *Kp*LPMO10A was evaluated after 0, 0.5, 1, 2, 4, 6, 8, 16 and 24 h at 37 °C/850 rpm. Experiments with H_2_O_2_ (0, 10, 25, 50, 75 and 100 μM) and CuSO_4_ (4.4 μM) in the absence of *Kp*LPMO10A were carried out to evaluate the occurrence of Fenton reactions using xylan as substrate.

The release of unoxidized and oxidized oligosaccharides was evaluated by high-performance anion-exchange chromatography coupled with pulsed electrochemical detection (HPAEC-PAD) following [58]. Peaks of native cello-/xylo-oligosaccharides (Glc_2_–Glc_6_/Xyl_3_–Xyl_6_) and cellobionic acid were assigned based on standards from Megazyme Inc. and Carbosynth, respectively. C1-oxidized xylo-oligosaccharides were produced by incubating Xyl_2_–Xyl_5_ (10 mM) with 2 μM cellobiose dehydrogenase (CDH) for 16 h at 50 °C and 850 rpm. All experiments were performed in triplicate.

### Detection of reaction products by MALDI-TOF MS

The analysis of products was carried out in a MALDI-TOF Autoflex Speed system (Bruker Corporation). One microliter of reaction was added to 1 µL of 2.5-dihydroxybenzoic acid (DHB) in TA30 (30% acetonitrile and 0.1% trifluoroacetic acid). A total of 1 µL was applied on GrounSteel plate and was evaluated in the mass range of 100–2500 Da. Data were acquired on positive polarity reflector mode. All analyses were carried out in comparison with a blank without the addition of *Kp*LPMO10A and only the peaks not found in the blank ones were considered.

### Saccharification assays

The synergy of *Kp*LPMO10A (1 mg/g substrate) and Celluclast 1.5 L (0.9 FPU/g substrate, Sigma-Aldrich) was evaluated. The reactions were carried out at 50 °C/200 rpm in 0.05 M sodium acetate buffer, pH 5.0 and 10 mg substrate for 24 h (Avicel^®^ and Filter Paper) or 72 h [alkaline-pretreated sugarcane bagasse (%); cellulose, 58.6 ± 1.2; hemicellulose, 22.1 ± 1.4 and lignin, 10 ± 0.4] in a total volume of 1 mL. Control reactions were carried out in the absence of *Kp*LPMO10A. The synergy of *Kp*LPMO10A (1 mg/g substrate) and LPMO9 (a fungal strictly cellulose-oxidizing LPMO [Corrêa et al. to be submitted], 1 mg/g substrate) with the xylanase *xyn*B/XAC4254 (0.5 mg/g substrate, [20]) on deconstruction of xylan from beechwood (10 mg) was evaluated in a 1 mL reaction containing 0.05 sodium phosphate buffer, pH 6.0, at 37 °C/1000 rpm. Ascorbic acid (1 mM) was added to some reactions. After the treatment, the samples were boiled for 10 min and centrifuged at 12,000*g*. The release of glucose/cellobiose/xylose and xylo-oligosaccharides was evaluated by high-performance liquid chromatography (HPLC) and HPAEC-PAD, respectively. All experiments were performed in triplicate.

### Bioinformatics analysis

All AA10 sequences already characterized were obtained from CAZy database (http://www.cazy.org). The catalytic domains were aligned using MEGA 7 [59] and clustering was performed in phyML 3.0 (http://www.atgc-montpellier.fr/phyml/, [60]). The final tree was obtained in iTOL (Interactive Tree of Life, https://itol.embl.de/), [61].

## Additional files

**Additional file 1: Table S1.** Primers used in this work.

**Additional file 2: Figure S1.** Substrate-binding assays. Eighty micrograms of *Kp*LPMO10A was incubated with Avicel^®^, PASC (A) and α-chitin (B) for 1 h (on ice) or 16 h (37 °C/850 rpm). The soluble, washed and insoluble fractions were monitored by SDS-PAGE. MM, molecular marker (Thermo Fischer Scientific); C, control without substrate; A, Avicel^®^; P, PASC; αC, α-chitin.

**Additional file 3: Figure S2.** HPAEC-PAD chromatograms. Enzymatic reactions with Avicel^®^ (A), xyloglucan (B), guar galactomannan (C) and konjac glucomannan (D) as substrates. Blank reactions were carried out with buffer, the respective substrate and ascorbic acid in the absence of *Kp*LPMO10A. nC, nanocoulomb

**Additional file 4: Figure S3.** MALDI-TOF MS of α- (A) and colloidal chitin (B). MALDI-TOF MS data confirmed the release of oxidized products from α- (A) and colloidal chitin (B) by *Kp*LPMO10A after 16 h at 37 °C. DP5, *m/z* 1056.281 (native); mono-sodiated lactone or ketoaldose, *m/z* 1054.276 (− 2 Da); mono-sodiated aldonic acid or gemdiol, *m/z* 1072.300 (+ 16 Da); di-sodiated adduct of aldonic acid, *m/z* 1094.300 (+ 38 Da). The peaks corresponding to the native species are higher than those from oxidized ones in experiments carried out with α-chitin given the contaminating chito-oligosaccharides in substrate samples. R. int., relative intensity.

**Additional file 5: Figure S4.** Evaluation of Fenton reactions by HPAEC-PAD. Reactions were carried out with 4.4 μM copper sulfate (CuSO_4_), the same concentration adopted for *Kp*LPMO10A in previous assays, along with hydrogen peroxide (0, 10, 25, 50, 75 and 100 μM), ascorbate (1 mM), buffer pH 6.0 and xylan from beechwood (2 mg/mL). No peaks related to C1-oxidized species were found. nC, nanocoulomb.

**Additional file 6: Figure S5.** Regioselectivity of products released by *Kp*LPMO10A from xylan from beechwood. The HPAEC-PAD profile of products released by *Kp*LPMO10A from xylan was compared with C1-oxidized xylo-oligosaccharides (XylXyl1A-Xyl_4_Xyl1A). No peaks corresponding to C1-oxidized species were found, suggesting that the oxidative cleavage of xylan by *Kp*LPMO10A occurs exclusively at C4. Xyl_3_–Xyl_5_ are native oligosaccharides. XylXyl1A–Xyl_4_Xyl1A correspond to aldonic acids. C1-oxidized xylo-oligosaccharides were produced upon the incubation of Xyl_3_–Xyl_5_ with cellobiose dehydrogenase (CDH). nC, nanocoulomb.

**Additional file 7: Figure S6.** Alignment of primary sequences of *Kp*LPMO10A (*K. papulosa*), *Sc*LPMO10B (*S. coelicolor*), E7 (*T. fusca*) and Micau_1230 (*M. aurantiaca*). S and H delimitations were based on *Kp*LPMO10A. Loop 2 is comprised between S1 and S3. S, strand; H, helix.

**Additional file 8: Figure S7.** Thermal shift assays of *Kp*LPMO10A at pH 5.0 (A) and 6.0 (B). *Kp*LPMO10A is the apo-enzyme. The assays *Kp*LPMO10A + Cu^++^ and *Kp*LPMO10A + Cu^++^ + EDTA were performed with the CuSO_4_-saturated enzyme. Thermal shift assays revealed the key role of CuSO_4_ for thermal stability of *Kp*LPMO10A. a.u., arbitrary unit.

**Additional file 9: Figure S8.** Overall structures (A, B and C) and substrate-binding surfaces (D, E and F) of the xylan-oxidizing *Kp*LPMO10A (A and D), *Ls*AA9A (*L. similis*, 5NLO, B and E) and *Pc*AA14A (*P. coccineus*, 5NO7, C and F). The catalytic residues (histidines) and the tyrosine responsible for the axial coordination of copper are highlighted. Loops are represented by dark colors. *Ls*AA9A is shown complexed with Xyl_5_. Only *Kp*LPMO10A and *Ls*AA9A cleave isolated xylan. *Pc*AA14A release oxidized products from xylan only when complexed with cellulose. No structural evidences were found for xylan-oxidizing capacity shown by these LPMOs.

**Additional file 10: Table S2.** Substrates evaluated for *Kp*LPMO10A activity.

## Abbreviations

LPMO: lytic polysaccharide monooxygenase
AA9: auxiliary activity 9
AA10: auxiliary activity 10
AA14: auxiliary activity 14
CAZy: carbohydrate active enzymes databank
CBP21: chitin-binding protein 21
CBM33: carbohydrate-binding module 33
pI: isoeletric point
PASC: phosphoric acid-swollen cellulose
C1: carbon 1
C4: carbon 4
AscAc: ascorbic acid
Glc: cello-oligosaccharide
GlcGlc1A: cellobionic acid
Xyl: xylo-oligosaccharide
CDH: cellobiose dehydrogenase
