# Supplementary material for: An actinobacteria lytic polysaccharide monooxygenase acts on both cellulose and xylan to boost biomass saccharification
Source: Biotechnol Biofuels. 2019 May 10;12:117. doi: 10.1186/s13068-019-1449-0 (PMC6509861; doi:10.1186/s13068-019-1449-0)
Supplement: Supplementary file 1 — Additional file 1: Table S1. Primers used in this work. [file 13068_2019_1449_MOESM1_ESM.docx]

**Additional file 1: Table S1 Primers used in this work**

| Name | Sequence (5’-3’) |
| --- | --- |
| KpF* | CACGGTTCCGTCGTCGAC |
| KpR | GGTGAAGTTCACGTCACTGCAC |
| KpFpET22b | CTGCCCAGCCGGCGATGGCCCACGGTTCCGTCGTCGAC |
| KpRpET22b | TCAGTGGTGGTGGTGGTGGTGGGTGAAGTTCACGTCACTGCAC |
| pET22bF | GCACACCACCACCACCACCA |
| pET22bR | GGCCATCGCCCGCTGGGCAC |

*The region corresponding to the signal peptide was excluded.

Underlined sequences have homology with pET-22b(+).
