# Supplementary material for: An actinobacteria lytic polysaccharide monooxygenase acts on both cellulose and xylan to boost biomass saccharification
Source: Biotechnol Biofuels. 2019 May 10;12:117. doi: 10.1186/s13068-019-1449-0 (PMC6509861; doi:10.1186/s13068-019-1449-0)
Supplement: Supplementary file 2 — Additional file 2: Figure S1. Substrate-binding assays. Eighty micrograms of KpLPMO10A was incubated with Avicel®, PASC (A) and α-chitin (B) for 1 h (on ice) or 16 h (37 °C/850 rpm). The soluble, washed and insoluble fractions were monitored by SDS-PAGE. MM, molecular marker (Thermo Fischer Scientific); C, control without substrate; A, Avicel®; P, PASC; αC, α-chitin. [file 13068_2019_1449_MOESM2_ESM.docx]

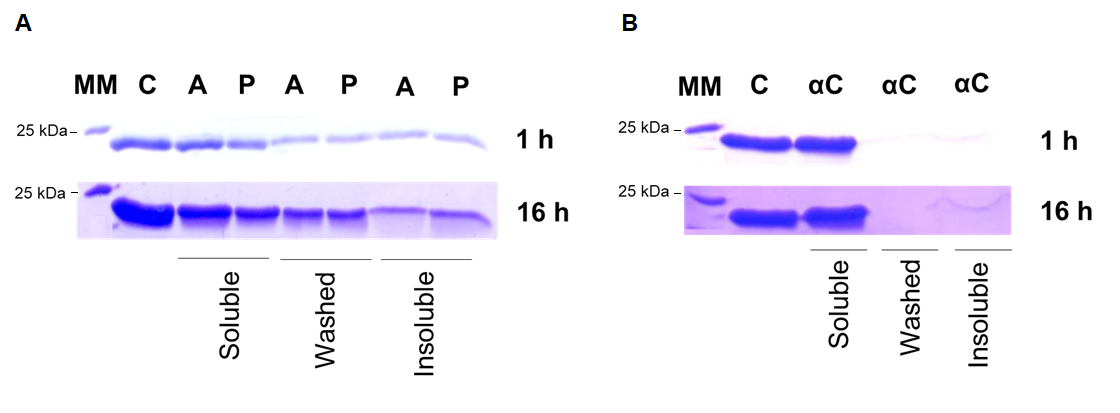


**Additional file 2: Figure S1 Substrate-binding assays.** Eighty micrograms of *Kp*LPMO10A was incubated with Avicel^®^, PASC (A) and α-chitin (B) during 1 h (on ice) or 16 h (37 ^o^C/850 rpm). The soluble, washed and insoluble fractions were monitored by SDS-PAGE. MM, molecular marker (Thermo Fischer Scientific); C, control without substrate; A, Avicel^®^; P, PASC; αC, α-chitin.
