# Supplementary material for: An actinobacteria lytic polysaccharide monooxygenase acts on both cellulose and xylan to boost biomass saccharification
Source: Biotechnol Biofuels. 2019 May 10;12:117. doi: 10.1186/s13068-019-1449-0 (PMC6509861; doi:10.1186/s13068-019-1449-0)
Supplement: Supplementary file 3 — Additional file 3: Figure S2. HPAEC-PAD chromatograms. Enzymatic reactions with Avicel® (A), xyloglucan (B), guar galactomannan (C) and konjac glucomannan (D) as substrates. Blank reactions were carried out with buffer, the respective substrate and ascorbic acid in the absence of KpLPMO10A. nC, nanocoulomb [file 13068_2019_1449_MOESM3_ESM.docx]

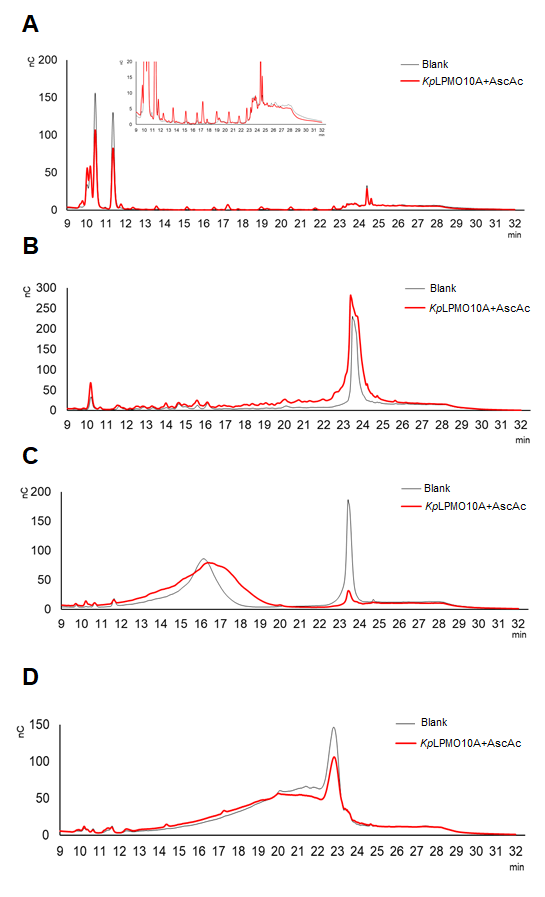


**Additional file 3: Figure S2 HPAEC-PAD chromatograms.** Enzymatic reactions with Avicel^®^ (A), xyloglucan (B), guar galactomannan (C) and konjac glucomannan (D) as substrates. Blank reactions were carried out with buffer, the respective substrate and ascorbic acid in the absence of *Kp*LPMO10A. nC, nanocoulomb.
