# Supplementary material for: An actinobacteria lytic polysaccharide monooxygenase acts on both cellulose and xylan to boost biomass saccharification
Source: Biotechnol Biofuels. 2019 May 10;12:117. doi: 10.1186/s13068-019-1449-0 (PMC6509861; doi:10.1186/s13068-019-1449-0)
Supplement: Supplementary file 4 — Additional file 4: Figure S3. MALDI-TOF MS of α- (A) and colloidal chitin (B). MALDI-TOF MS data confirmed the release of oxidized products from α- (A) and colloidal chitin (B) by KpLPMO10A after 16 h at 37 °C. DP5, m/z 1056.281 (native); mono-sodiated lactone or ketoaldose, m/z 1054.276 (− 2 Da); mono-sodiated aldonic acid or gemdiol, m/z 1072.300 (+ 16 Da); di-sodiated adduct of aldonic acid, m/z 1094.300 (+ 38 Da). The peaks corresponding to the native species are higher than those from oxidized ones in experiments carried out with α-chitin given the contaminating chito-oligosaccharides in substrate samples. R. int., relative intensity. [file 13068_2019_1449_MOESM4_ESM.docx]

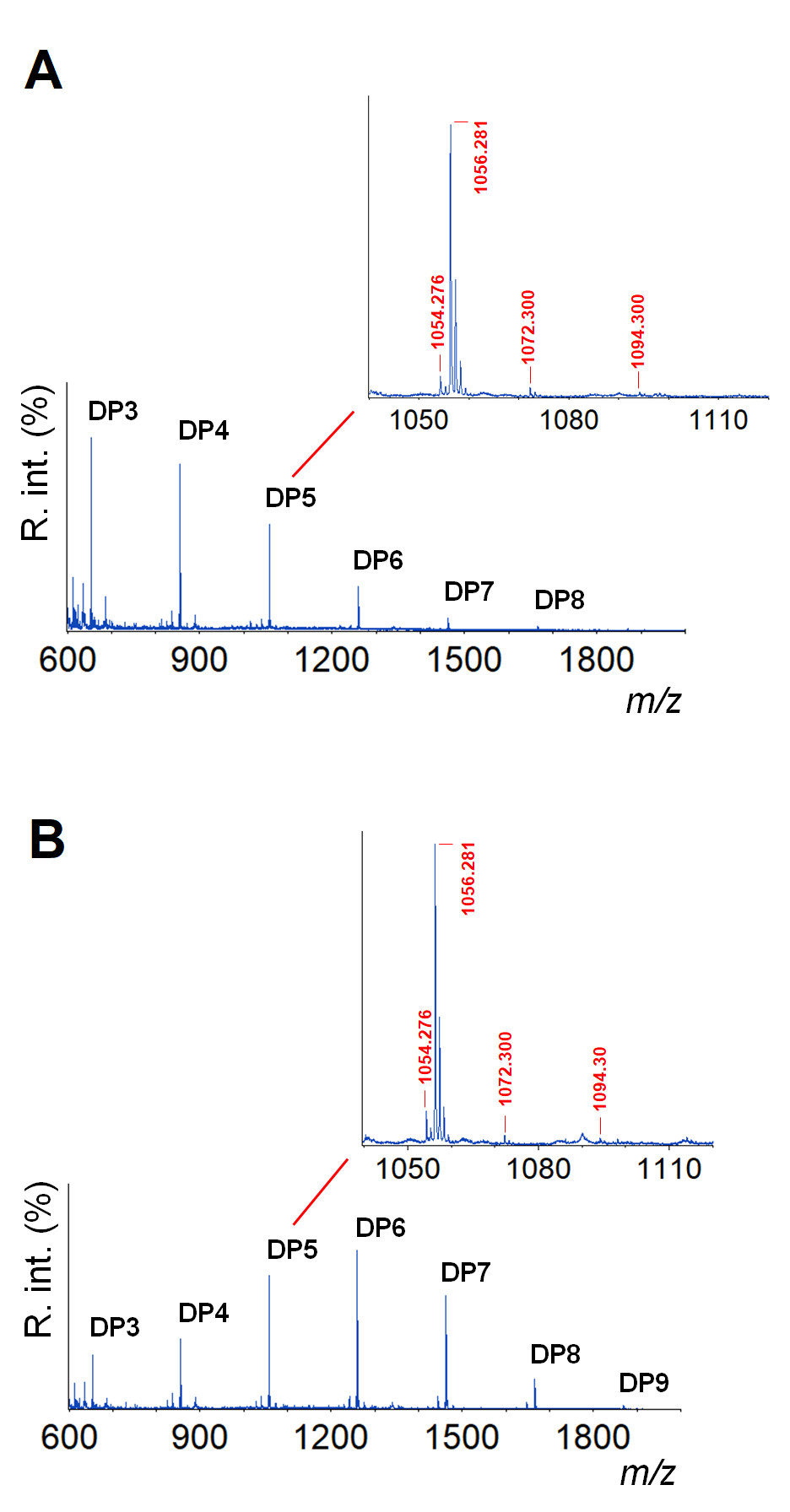


**Additional file 4: Figure S3 MALDI-TOF MS of α- (A) and colloidal chitin (B).** MALDI-TOF MS data confirmed the release of oxidized products from α- (A) and colloidal chitin (B) by *Kp*LPMO10A after 16 h at 37 ^o^C. DP5, *m/z* 1056.281 (native); mono-sodiated lactone or ketoaldose, *m/z* 1054.276 (-2 Da); mono-sodiated aldonic acid or gemdiol, *m/z* 1072.300 (+16 Da); di-sodiated adduct of aldonic acid, *m/z* 1094.300 (+38 Da). The peaks corresponding to the native species are higher than those from oxidized ones in experiments carried out with α-chitin given the contaminating chito-oligosaccharides in substrate samples. R. int., relative intensity.
