# Supplementary material for: An actinobacteria lytic polysaccharide monooxygenase acts on both cellulose and xylan to boost biomass saccharification
Source: Biotechnol Biofuels. 2019 May 10;12:117. doi: 10.1186/s13068-019-1449-0 (PMC6509861; doi:10.1186/s13068-019-1449-0)
Supplement: Supplementary file 5 — Additional file 5: Figure S4. Evaluation of Fenton reactions by HPAEC-PAD. Reactions were carried out with 4.4 μM copper sulfate (CuSO4), the same concentration adopted for KpLPMO10A in previous assays, along with hydrogen peroxide (0, 10, 25, 50, 75 and 100 μM), ascorbate (1 mM), buffer pH 6.0 and xylan from beechwood (2 mg/mL). No peaks related to C1-oxidized species were found. nC, nanocoulomb. [file 13068_2019_1449_MOESM5_ESM.docx]

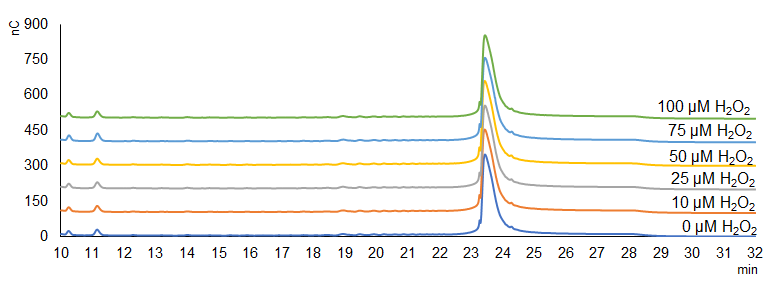


**Additional file 5: Figure S4 Evaluation of Fenton reactions by HPAEC-PAD.** Reactions were carried out with 4.4 μM copper sulfate (CuSO_4_), the same concentration adopted for *Kp*LPMO10A in previous assays, along with hydrogen peroxide (0, 10, 25, 50, 75 and 100 μM), ascorbate (1 μM), buffer pH 6.0 and xylan from beechwood (2 mg/mL). No peaks related to C1-oxidized species were found. nC, nanocoulomb.
