# Supplementary material for: An actinobacteria lytic polysaccharide monooxygenase acts on both cellulose and xylan to boost biomass saccharification
Source: Biotechnol Biofuels. 2019 May 10;12:117. doi: 10.1186/s13068-019-1449-0 (PMC6509861; doi:10.1186/s13068-019-1449-0)
Supplement: Supplementary file 6 — Additional file 6: Figure S5. Regioselectivity of products released by KpLPMO10A from xylan from beechwood. The HPAEC-PAD profile of products released by KpLPMO10A from xylan was compared with C1-oxidized xylo-oligosaccharides (XylXyl1A-Xyl4Xyl1A). No peaks corresponding to C1-oxidized species were found, suggesting that the oxidative cleavage of xylan by KpLPMO10A occurs exclusively at C4. Xyl3–Xyl5 are native oligosaccharides. XylXyl1A–Xyl4Xyl1A correspond to aldonic acids. C1-oxidized xylo-oligosaccharides were produced upon the incubation of Xyl3–Xyl5 with cellobiose dehydrogenase (CDH). nC, nanocoulomb. [file 13068_2019_1449_MOESM6_ESM.docx]

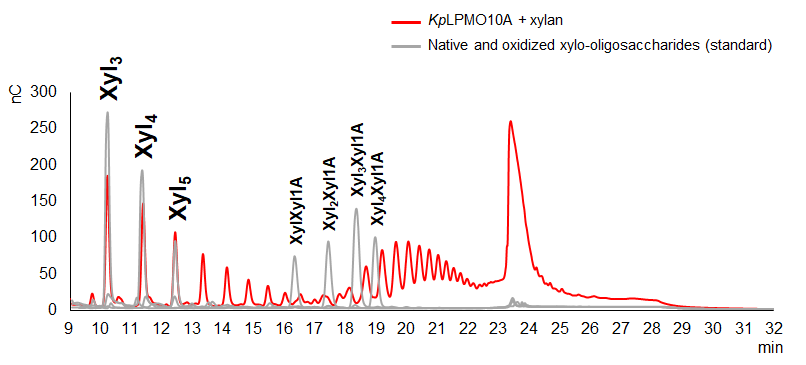


**Additional file 6: Figure S5 Regioseletivity of products released by *Kp*LPMO10A from beechwood xylan.** The HPAEC-PAD profile of products released by *Kp*LPMO10A from xylan was compared with C1-oxidized xylo-oligosaccharides (XylXyl1A-Xyl_4_Xyl1A). No peaks corresponding to C1-oxidized species were found, suggesting that the oxidative cleavage of xylan by *Kp*LPMO10A occurs exclusively at C4. Xyl_3_-Xyl_5_ are native oligosaccharides. XylXyl1A-Xyl_4_Xyl1A correspond to aldonic acids. C1-oxidized xylo-oligosaccharides were produced upon the incubation of Xyl_2_-Xyl_5_ with cellobiose dehydrogenase (CDH). nC, nanocoulomb.
