# Supplementary material for: An actinobacteria lytic polysaccharide monooxygenase acts on both cellulose and xylan to boost biomass saccharification
Source: Biotechnol Biofuels. 2019 May 10;12:117. doi: 10.1186/s13068-019-1449-0 (PMC6509861; doi:10.1186/s13068-019-1449-0)
Supplement: Supplementary file 7 — Additional file 7: Figure S6. Alignment of primary sequences of KpLPMO10A (K. papulosa), ScLPMO10B (S. coelicolor), E7 (T. fusca) and Micau_1230 (M. aurantiaca). S and H delimitations were based on KpLPMO10A. Loop 2 is comprised between S1 and S3. S, strand; H, helix. [file 13068_2019_1449_MOESM7_ESM.docx]

**
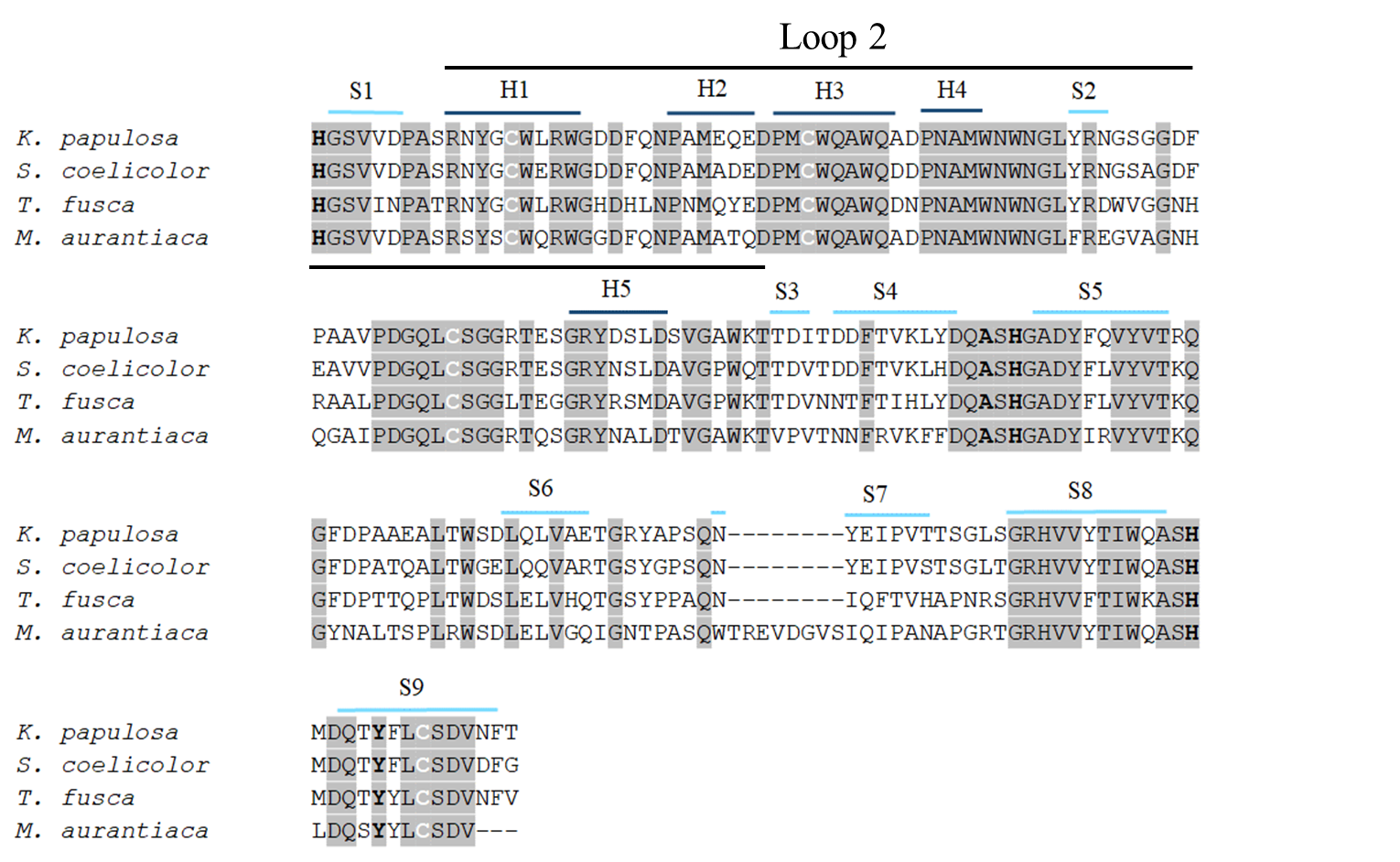
**

**Additional file 7: Figure S6: Alignment of primary sequences of *Kp*LPMO10A (*K.papulosa*), *Sc*LPMO10B (*S. coelicolor*), E7 (*T. fusca*) and Micau_1230 (*M. aurantiaca*).** S and H delimitations were based on *Kp*LPMO10A. Loop 2 is comprised between S1 and S3. S, strand; H, helix.
