# Supplementary material for: An actinobacteria lytic polysaccharide monooxygenase acts on both cellulose and xylan to boost biomass saccharification
Source: Biotechnol Biofuels. 2019 May 10;12:117. doi: 10.1186/s13068-019-1449-0 (PMC6509861; doi:10.1186/s13068-019-1449-0)
Supplement: Supplementary file 8 — Additional file 8: Figure S7. Thermal shift assays of KpLPMO10A at pH 5.0 (A) and 6.0 (B). KpLPMO10A is the apo-enzyme. The assays KpLPMO10A + Cu++ and KpLPMO10A + Cu++ + EDTA were performed with the CuSO4-saturated enzyme. Thermal shift assays revealed the key role of CuSO4 for thermal stability of KpLPMO10A. a.u., arbitrary unit. [file 13068_2019_1449_MOESM8_ESM.docx]

**
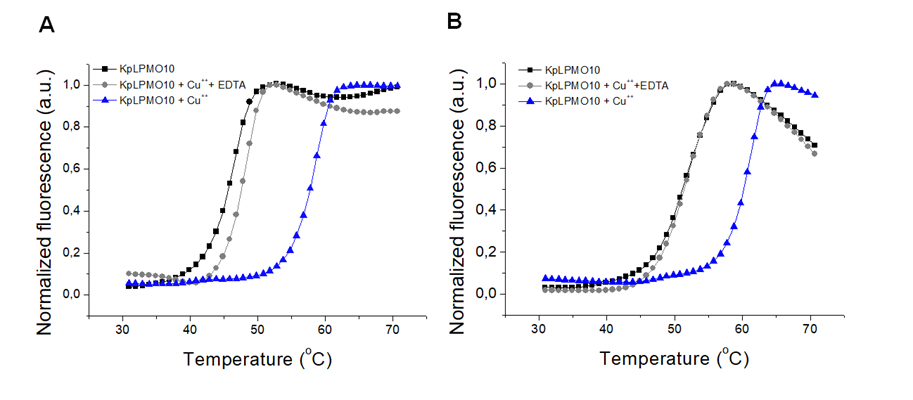
**

**Additional file 8: Figure S7 Thermal Shift assays of *Kp*LPMO10A at pH 5.0 (A) and 6.0 (B).** *Kp*LPMO10A is the apo-enzyme. The assays *Kp*LPMO10A + Cu^++^ and *Kp*LPMO10A + Cu^++^ + EDTA were performed with the CuSO_4_-saturated enzyme. Thermal shift assays revealed the key role of CuSO_4_ for thermal stability of *Kp*LPMO10A. a.u., arbitrary unit.
