# Supplementary material for: An actinobacteria lytic polysaccharide monooxygenase acts on both cellulose and xylan to boost biomass saccharification
Source: Biotechnol Biofuels. 2019 May 10;12:117. doi: 10.1186/s13068-019-1449-0 (PMC6509861; doi:10.1186/s13068-019-1449-0)
Supplement: Supplementary file 9 — Additional file 9: Figure S8. Overall structures (A, B and C) and substrate-binding surfaces (D, E and F) of the xylan-oxidizing KpLPMO10A (A and D), LsAA9A (L. similis, 5NLO, B and E) and PcAA14A (P. coccineus, 5NO7, C and F). The catalytic residues (histidines) and the tyrosine responsible for the axial coordination of copper are highlighted. Loops are represented by dark colors. LsAA9A is shown complexed with Xyl5. Only KpLPMO10A and LsAA9A cleave isolated xylan. PcAA14A release oxidized products from xylan only when complexed with cellulose. No structural evidences were found for xylan-oxidizing capacity shown by these LPMOs. [file 13068_2019_1449_MOESM9_ESM.docx]

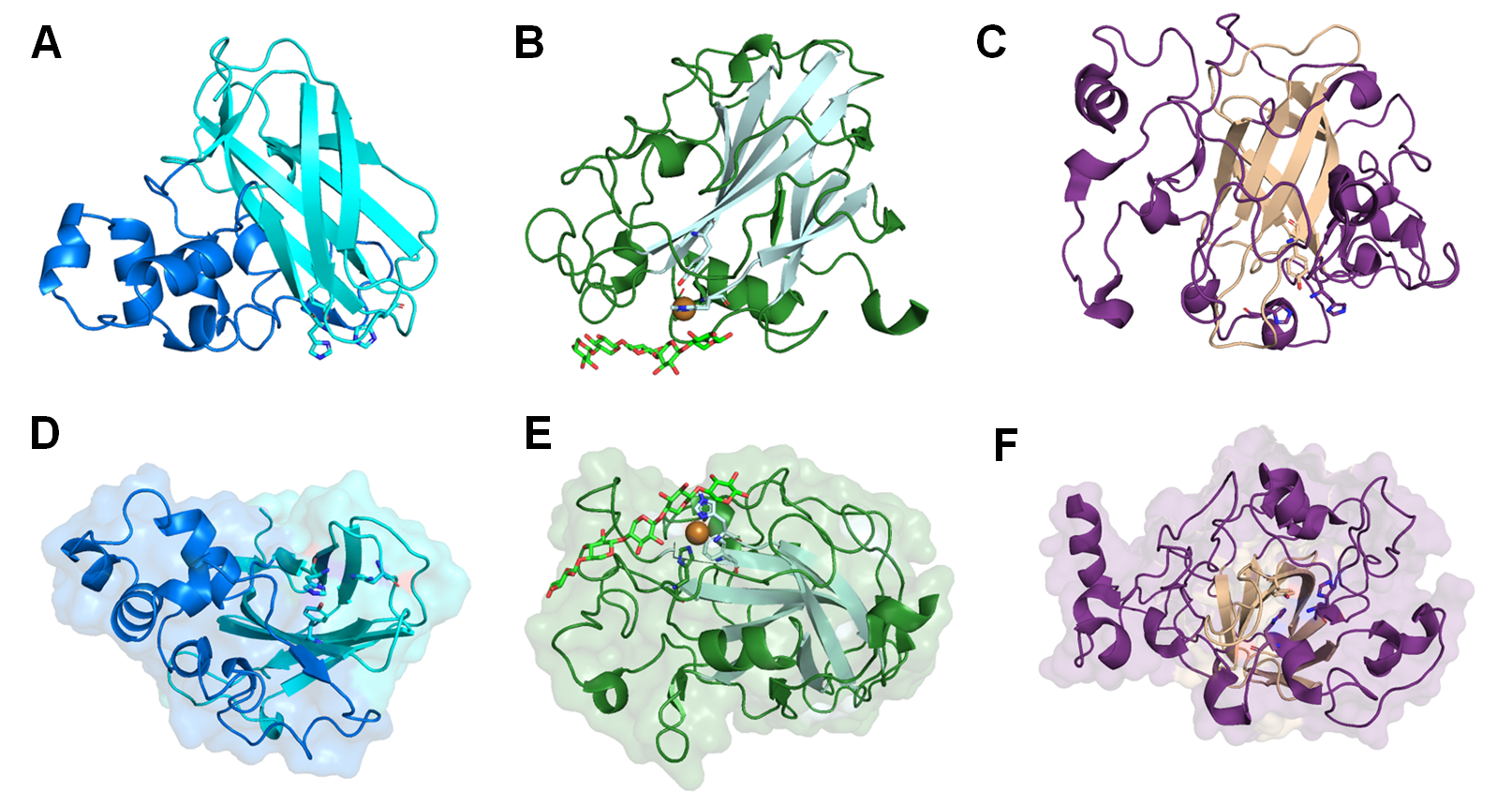


**Additional file 9: Figure S8 Overall structures (A, B and C) and substrate binding surfaces (D, E and F) of the xylan oxidizing *Kp*LPMO10A (A and D), *Ls*AA9A (*L. similis*, 5NLO, B and E) and *Pc*AA14A (*P. coccineus*, 5NO7, C and F).** The catalytic residues (histidines) and the tyrosine responsible for the axial coordination of copper are highlighted. Loops are represented by dark colors. *Ls*AA9A is shown complexed with Xyl_5_. Only *Kp*LPMO10A and *Ls*AA9A cleave isolated xylan. *Pc*AA14A release oxidized products from xylan only when complexed with cellulose. No structural evidences were found for xylan-oxidizing capacity shown by these LPMOs.
