# Supplementary material for: An actinobacteria lytic polysaccharide monooxygenase acts on both cellulose and xylan to boost biomass saccharification
Source: Biotechnol Biofuels. 2019 May 10;12:117. doi: 10.1186/s13068-019-1449-0 (PMC6509861; doi:10.1186/s13068-019-1449-0)
Supplement: Supplementary file 10 — Additional file 10: Table S2. Substrates evaluated for KpLPMO10A activity. [file 13068_2019_1449_MOESM10_ESM.docx]

**Additional file 10: Table S2.** Substrates evaluated for *Kp*LPMO10A activity

| **Substrate** | **Activity** |
| --- | --- |
| α-chitin | + |
| α-chitin treated with Phosphoric Acid | + |
| Phosphoric Acid Swollen Cellulose - PASC | + |
| Avicel^®^ | + |
| Cellopentaose | - |
| Cellohexaose | - |
| Xylan from Beechwood | + |
| Konjac Glucomannan | - |
| Guar Galactomannan | - |
| Xyloglucan | - |
| Xylopentaose | - |
| Xylohexaose | - |

+, enzymatic activity recorded; -, no enzymatic activity recorded.
